# 

**Published:** 1889-05-25

**Authors:** 


### NOTICE TO CORRESPONDENTS.

All MS., Letters, Books for Review, and other matters intended for the Editor, should be addressed The Editor, The Lodge, Porchester Square, London, W.

### Notice to Advertisers and Subscribers.

All Advertisements, Orders for Copies of The Hospital, and Business Communications should be addressed to The Publisher, *not to the Editor* The Hospital, 139-140, Salisbury-court, Fleet-street, E.C.

Vol. V., now ready, 4s., post free. Cases for binding, may be had of the Publisher, at 1s. 6d. each.

### To Residents, Secretaries, and Matrons.

The Editor will be glad to have the Regulations and each Report as issued by Asylums, Hospitals, Convalescent and Nursing Institutions, as well as those of other Charities, and an early copy in duplicate of all Appeals and Festival Notices, etc., addressed to *The Editor, The Lodge, Porchester-square, London, W.* Any superintendent, secretary, matron, or other chief official who regularly sends such information may be registered, on application to the Publisher, to receive free of cost a cover for binding The Hospital in half-yearly volumes.

### Amusements and Relaxation.

### Word Competition.

Commenced April 6th, Ends June 29th.

Three Prizes of 15s., 10s., 5s., will be given for the largest number of words derived from the words set for dissection.

Proper names, abbreviations, foreign words, words of less than four letters, and repetitions are barred; plurals, and past and present participles of verbs, are allowed. Nuttall's *Standard* dictionary only to be used.

N.B.—Word dissections must be sent in WEEKLY, arranged alphabetically, with correct total affixed.

The word for dissection for this, the *Eighth* week of the quarter, being "ROSEBERRY."

### Results of Sixth Week.

| Names. | May 16th. | Totals. | Names. | May 16th. | Totals. |
|---|---|---|---|---|---|
| Patience | 18 | 520 | Tiny | 18 | 431 |
| Nurse Duty | 18 | 498 | N'importe Qui | 16 | 405 |
| Lightowlers | 18 | 498 | Pollie | 18 | 379 |
| Scrooge | 18 | 479 | W. G. R. | 17 | 377 |
| Reldas | — | 250 | Robe | — | 150 |
| Tinie | 18 | 523 | Beryl | 14 | 352 |
| K. G. | 17 | 480 | Nesta | — | 124 |
| Broxbourne | 18 | 487 | Daffodil | — | 89 |
| Lauriston | 18 | 490 | A Schoolgirl | 13 | 143 |
| Spes | 18 | 486 | Montague | — | 79 |
| Gretta | — | 224 | Sheeny | — | 111 |
| Francesca | 17 | 476 | Alice R. | — | 63 |
| Heatherbell | — | 185 | Belfast | — | 163 |
| Sycamore | — | 201 | Gentian | — | 209 |
| Arctus | 15 | 421 | Hope | — | 39 |
| Amicus | 18 | 486 | Louie | — | 139 |
| Scratcher | — | 197 | Hamilton | — | 121 |
| Jemima | — | 138 | E. M. | — | 120 |
| Concours | 17 | 427 | F. J. D. | — | 112 |
| C. J. S. | — | 134 | Ladyr | — | 102 |
| See-Saw | 17 | 434 | Surgical Nurse | 13 | 128 |
| Rosemont | — | 173 | Violet | — | 95 |
| Jenny Wren | 17 | 262 | L. Pearce | — | 32 |
| Eric | — | 402 | Contentio | 17 | 98 |

### Notice to all Correspondents.

N.B.—All letters referring to this page which do not arrive at 139 and 140, Salisbury-court, London, E.C., by the *first post on Thursdays*, and are not addressed PRIZE EDITOR, will in future be disqualified and disregarded.

### Conditions.

I. Every paper sent in must be clearly written, and one side only must be used. All competitors must mark at the head of their papers the number of their competition.

II. Each paper must be signed by the author with his or her real name and address. A *nom de plume* may be added if the writer does not desire to be referred to by us by his real name. In the case of all prize-winners, however, the real name and address will be published.

III. All communications relating to prize competitions should be addressed to "The Prize Editor, The Hospital, 139 and 140, Salisbury-court, London, E.C." Competitors are requested not to include in letters, etc., to the Prize Editor communications on matters of other business. All letters must be fully prepaid.

IV. The Prize Editor of The Hospital is the sole judge of the competitions, and his decision is in all cases final and without appeal.

V. The Prize Editor reserves the right to publish any paper sent in, whether it receives a prize or not. He does not hold himself responsible for any MSS. sent, nor can he undertake to return rejected competitions.

### Scraps and Gleanings.

The Lord Mayor visited the Pasteur Institute while at Paris.

Surgeon Martin Gaysford died lately at Kathgodan from cholera.

Mr. W. E. Wynter has been appointed registrar of Middlesex Hospital.

The late Alderman Goldschmidt has left £100 to the Manchester Royal Eye Hospital.

M. Pasteur will deliver the Croonian lecture on June 23rd, if his health permits.

An effort is being made to open a cottage hospital at Shanklin for the cure of consumption.

Dr. Murphy, of Worcester, has been presented with a writing table by his ambulance pupils.

Messrs. Barraud, of Oxford-street, have published three photographs of the late Father Damien.

Prince Joseph Sulkowski, who escaped from a lunatic asylum near Vienna, has been recaptured.

Huntly Jubilee Cottage Hospital will be opened by the Duke of Richmond and Gordon in September next.

Dr. Crowe has been appointed first assistant medical officer at Chorley, in place of the late Dr. Mason.

The Duchess of Albany opened the sale of work at Kensington Town Hall, in aid of French Charities in London.

The first meeting has been held of the Salford Working Men's Sanitary Association. The Rev. H. T. Smart is chairman.

Devonshire Hospital, Buxton, treated 2,642 patients last year, of which over a thousand were cases of rheumatism.

Miss Lilias Graham, of the Mission Hospital at Amoy, China, appeals in the *Morning Post* for subscriptions and old linen.

The Sultan of Turkey has granted 500,000 piastres for the erection of new laboratories for the Medical School at Constantinople.

Mrs. Dumas, of Bromley, has gone to Paris in order to undergo M. Pasteur's treatment for hydrophobia, through the bite of a dog.

Last Saturday the presentation was made of the life-governorship of the East London Working Men's Committee to the Lady Mayoress.

The weavers of Lancashire are raising a memorial to the late Dr. Stephenson, who agitated against excessive steaming in weaving-sheds.

Malvern Provident Dispensary has 314 members on its books. The annual subscriptions amount to £76, and there is a deficiency of £48 to be met.

The West Ham and South Essex Dispensary issues a satisfactory report. The Clothworkers' Company is particularly generous to this charity.

Mrs. Scharlieb, M.D., received her medical degree at London University last week, being the first woman who has achieved this triumph.

Mr. Edward B. Hill, B.A.Cantab., M.R.C.S.E., L.S.A., has been appointed house surgeon at Richmond Hospital, vice Mr. Pedler, resigned.

The Queen sent a message, in which she wished success to the Mission to Deep-Sea Fishermen, on the occasion of the May meeting in support of that mission.

Spennymoor needs a cottage hospital. A child was terribly injured there by a roundabout lately and had to be taken to Newcastle, a distance of twenty miles.

The death is announced of Lord Sidney Godolphin Osborn, whose pamphlet of "The Hospitals of Scutari" received much notice at the time of the Crimean War.

The late Mrs. Sargent, daughter of O. W. Holmes, has left £2,000 to the anatomical department of Harvard College, and £1,000 to the Massachusetts General Hospital.

Dr. Paget, second surviving son of Sir George E. Paget, has been appointed medical officer of health for Salford. One more of a famous family likely to come to the front in medical science.

Miss N. M. Dean, a graduate in medicine of Wisconsin University, has been in successful practice at Helena, Montana, for about four years, and her income last year is said to have reached over £2,000.

At Epsom, on May 27th, Mr. Arthur Thompson, Professor of the Guildhall School of Music, will give a musical entertainment in aid of the funds of the new cottage hospital, assisted by some of his pupils.

The Yorkshire Rugby Football Union, at a meeting held in Leeds a few days ago, voted the sum of £903 10s. out of the proceeds of the challenge cup competition to the county medical and charitable institutions.

The death of the Rev. J. St. John Blunt vacates the post of Master to St. Katherine's Hospital, worth £2,000 a year. After the public attention called to the affairs of this hospital in connection with the Jubilee Fund, it is possible the post may not be refilled.

A festival service will be held in the nave of Westminster Abbey on Ascension Day, 30th May, at 7 p.m., in aid of Westminster Hospital. The service will include Mendelssohn's oratorio, "Elijah," with full orchestra and chorus numbering nearly 400 performers.

Mr. A. Thorndyke Rice, who was recently appointed United States Minister to Russia, is reported to have died suddenly. Mr. Rice had been suffering from quinsy, but his condition was not considered dangerous. Suddenly, however, œdema of the glottis set in and stopped respiration.

The Church of England Temperance Society has received a donation of £100 from an anonymous donor to provide a missionary for Clerkenwell Police-court. A similar sum has also been promised by the Rev. Dr. Oliver, of St. Mary's, Ealing, for a missionary for the Thames Police-court.

On the motion of Mr. Brudenell Carter, the London County Council has appointed a committee "to enquire into and to report to the Council upon the advantages which might be expected from the establishment, as a complement to the existing asylum system, of a hospital, with a visiting medical staff, for the study and curative treatment of insanity."

